# Non-Communication Decentralized Multi-Robot Collision Avoidance in Grid Map Workspace with Double Deep Q-Network

**DOI:** 10.3390/s21030841

**Published:** 2021-01-27

**Authors:** Lin Chen, Yongting Zhao, Huanjun Zhao, Bin Zheng

**Affiliations:** 1 Chongqing Institute of Green and Intelligent Technology, Chinese Academy of Sciences, Chongqing 400700, China; chenlin18@cigit.ac.cn (L.C.); zhaoyongting@cigit.ac.cn (Y.Z.); zhaohuanjun@cigit.ac.cn (H.Z.); 2 School of Computer Science and Technology, University of Chinese Academy of Sciences, Beijing 100049, China

**Keywords:** robot learning, deep reinforcement learning, grid map workspace

## Abstract

This paper presents a novel decentralized multi-robot collision avoidance method with deep reinforcement learning, which is not only suitable for the large-scale grid map workspace multi-robot system, but also directly processes Lidar signals instead of communicating between the robots. According to the particularity of the workspace, we handcrafted a reward function, which considers both the collision avoidance among the robots and as little as possible change of direction of the robots during driving. Using Double Deep Q-Network (DDQN), the policy was trained in the simulation grid map workspace. By designing experiments, we demonstrated that the learned policy can guide the robot well to effectively travel from the initial position to the goal position in the grid map workspace and to avoid collisions with others while driving.

## 1. Introduction

Multi-robot navigation is widely used in multi-robot search and rescue, autonomous warehouses, intelligent robot systems for sorting, navigation through human crowds, and other fields. With the development of robotics and artificial intelligence, researchers [1,2,3] have studied how to apply advanced algorithms in artificial intelligence to multi-robot navigation. The core part of multi-robot navigation is to make the robot travel from the initial position to the target position efficiently, avoiding collision with others [4].

**Figure 1:**
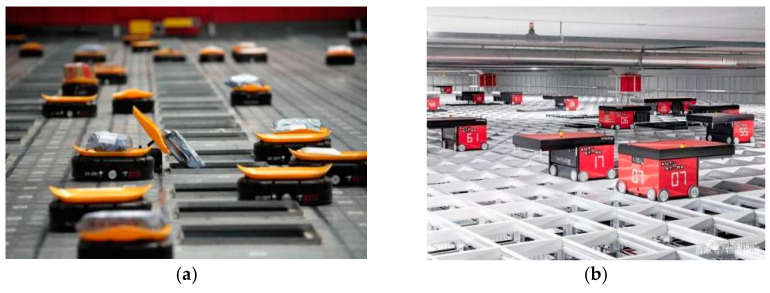
Grid map workspace application scenarios: (**a**) a sorting robot system, cited in web (http://www.sd.chinanews.com/2/2018/0606/59838.html), (**b**) an autonomous warehouse, cited on the web (https://baijiahao.baidu.com/s?id=1637276211094569677&wfr=spider&for=pc).

**Figure 2:**
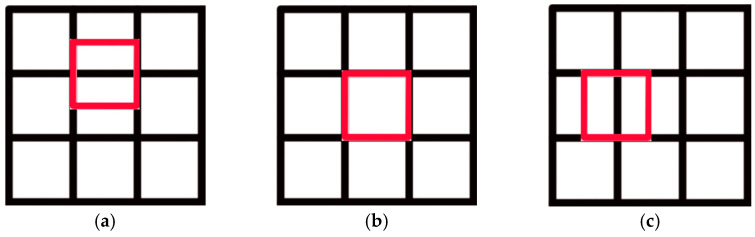
Schematic diagram of the constraints in the grid map workspace: (**a**–**c**) the three states of the robot working in the grid map workspace.

Researchers [1,2,3,4,5,6,7,8] have studied the decentralized multi-robot collision avoidance algorithm and some fruitful results have been achieved, such as collision avoidance with deep reinforcement learning (CADRL) [1], socially aware CADRL (SA-CADRL) [2], reciprocal velocity obstacle (RVO) [5]. The methods mentioned above were designed for cluttered workspaces. In all positions in an obstacle-free environment, the robot can move in any direction. In the real world, there are scenes where multiple robots work in a grid map workspace, such as autonomous warehouses and sorting robot systems (Figure 1a). However, the grid map workspace has constraints on robot behavior (Figure 2). When the robot is in the state shown in Figure 2a, it can only move forward and backward. When it is in the state shown in Figure 2b, the robot can only move forward, backward, walk left, walk right, and stop. When the robot is in the state shown in Figure 2c, it can only walk left and right. Therefore, the decentralized method mentioned above is not suitable for the grid map workspace.

On the contrary, centralized methods [9,10,11,12,13] have been explored by some researchers, assuming that a central server fully understands the workspace information and the environmental perception information around each controlled robot. Using a planning algorithm, the central server produces a path that allows the robot to avoid collisions with others. Most of these methods are applicable to grid map workspaces. However, these methods heavily rely on the communication network between the central server and the robot [8]. Moreover, they can be computationally prohibitive for large multi-robot systems [14]. If the central server and/or the communication network crashes, the multi-robot system will be paralyzed.

We developed a novel decentralized multi-robot collision avoidance method with deep reinforcement learning, which is not only suitable for the large-scale grid map workspace multi-robot system, but also directly processes Lidar signals instead of communicating between the robots.

The related work of multi-robot collision avoidance policy is outlined in Section 2. Section 3 shows the mathematical framework of the problem. The reinforcement learning framework is described in detail in Section 4. The process of designing experiments and verifying results is explained in Section 5. Finally, we provide the conclusion in Section 6.

## 2. Related Work

Many researchers have conducted extensive research on the problem of multi-robot motion planning, achieving some good results. Some of the work related to this problem has been reviewed in paper [15,16]. The research can be classified into centralized methods [17,18,19] and decentralized methods [20,21,22,23]. Assuming that information about the position, velocity, and target position of all agents can be obtained, centralized methods treat the motion planning problem as an optimization problem. The former approach includes four categories: A* search expansion [24,25], increasing cost tree search [26], conflict-based search [27,28], and protocol-based [9]. The goal of optimization is to guide all agents to their target positions, while avoiding collisions with one another and minimizing goals such as energy or time. Augugliaro et al. [18] regarded the problem of multi-robot motion planning as a non-convex optimization problem, which can be solved using continuous convex programming. Based on linear programming, a centralized algorithm was proposed by Yu et al. in [9]. The purpose was to minimize the arrival time of the last agent, the maximum (single-agent) traveled distance, the total arrival time, and the total distance. Tang et al. [17] divided the problem into two stages to solve it. First, a geometric algorithm was used to find the piecewise linear trajectories of each robot, and then these trajectories were refined into high-order piecewise polynomials. However, for solving a large-scale optimization problem, computational complexity of centralized methods is inevitable as the number of agents increases. In addition, centralized methods rely heavily on the communication between the central server and the agent. Before this, many researchers have conducted a lot of work on decentralized algorithms and have achieved some good results. The decentralized methods are divided into learning-based methods [1,2,3,4] and traditional methods [5,20,29,30,31]. Traditional methods were discussed in paper [15], such as reciprocal velocity obstacle (RVO) [5] and optimal reciprocal collision avoidance (ORCA) [20]. Assuming that each agent has perfect knowledge about its neighbor’s shape, position, and velocity, the agent uses the optimal reciprocal collision avoidance (ORCA) [20] algorithm to calculate a velocity to keep it safe over the next time horizon.

The learning-based method, which benefits from advances in machine learning technology, is considered to be a promising direction to solve the problem. Some researchers [1,2,8] have formulated the multi-agent collision avoidance problem as a sequential decision-making problem in the reinforcement learning framework. The CADRL algorithm was proposed by Chen et al. in [1] based on a deep reinforcement learning framework, which offloads expensive real-time motion planning calculations to the offline training process. Long et al. in [4] developed a deep reinforcement learning framework. By training this framework, a decentralized sensor-level collision avoidance network can be obtained. The steering command of each agent can be calculated by feeding the raw Lidar sensor data to the network. Everett et al. in [8] proposed the GA3C-CADRL (GPU/CPU Asynchronous Advantage Actor-Critic for Collision Avoidance with Deep reinforcement learning) algorithm, which showed good performance for solving path planning problems. However, the decentralized methods mentioned above are only applicable to cluttered workspaces and cannot be used in grid map workspaces. We developed a novel deep reinforcement learning framework that can learn a decentralized collision avoidance policy in the grid map workspace. It was proved by experiments that agents can easily avoid collisions with others and can effectively complete tasks in the grid map workspace.

## 3. Problem Formulation

This section introduces the mathematical framework for collision avoidance among robots. All robots were modeled as squares with side length *D* working in a grid workspace. The problem could be treated as *N* robots moving in the grid map workspace. The robot could move one grid distance at a constant speed *v* or stay in the original position (center of the grid) within each time period *T*.

The core of the problem was to find a policy that could guide the robot to travel from the initial position to the target position, to avoid collisions with others during driving, and to minimize the number of direction changes during the completion of the task. Reducing the direction during driving can reduce the running time of the robot in reality.

In this scenario, at time *kT* (*k* = 0, 1, 2, …), each robot *i* in state *s_i_^kT^* took an action *a_i_^kT^* according to the policy *π* that drove the robot from the current position *p_i_^kT^* to the goal *g_i_* while avoiding collision with others. State *s^kT^* was composed of three parts (Equation (1)): state *s_o_^kT^* (*s_o_^kT^* ∈ *R*3 × 128) contained a total of 3 × 128 data, which were composed of the distance data scanned by the Lidar sensor (which was placed at the center of the robot to obtain 128 distance data of the 360-degree environment around the robot) at times (*k* − 2)*T*, (*k* − 1)*T*, and *kT*, respectively; *s_g_^kT^* was the relative target position of the robot, and *s_a_^kT^* represented the last action performed by the robot. Table 1 shows the correspondence between *s_a_^kT^* and the last action.

(1) $$s^{kT}=\{s_{o}^{kT},s_{g}^{kT},s_{a}^{kT}\}$$

There were five choices of action *a^kT^* (move forward, move backward, move left, move right, or stop) for the robot to perform at time *kT* during the movement. It was assumed that the robot needed time *T* to travel from the center position of the grid at time *kT* to the central location of the adjacent grid at a constant speed *v*. The action space, which contained five elements, was therefore finite. The set *As* of the action space was expressed as follows:

(2) As={(v,0),(−v,0),(0,v),(0,−v),(0,0)}

After collecting the data of *s^kT^*, the robot calculated the action instructions *a^kT^* based on the learned policy *π*:

(3) $$a^{kT}\sim \pi_{\theta }(a^{kT}|s^{kT}),k=0,1,2\ldots$$

where *θ* refers to the parameters in the policy model. We assumed that the last action of the robot at the initial position was stop, with *l_i_* representing the path of the *i*-th robot, and the set of *N* robot paths was expressed as follows:

(4) $$\begin{array}{l} L=\{l_{i},i=1,2,\ldots ,N|\\ a_{i}^{kT}\sim \pi_{\theta }(a_{i}^{kT}|s_{i}^{kT}),\\ p_{i}^{kT}=p_{i}^{(k-1)T}+T\cdot a_{i}^{(k-1)T}\\ k=0,1,2,\ldots ,\;p_{i}=[p_{ix},p_{iy}],\\ \forall j\in [1,N],\;j\neq i:\\ \left\|p_{ix}-p_{jx}\right\|+\left\|p_{iy}-p_{jy}\right\|\geq D\} \end{array}$$

where *p_i_* represents the position of the *i*-th robot.

The *i*-th robot needed *k_i_^g^T* time to travel from its initial position to the goal position. We expected all robots to use the same policy *π_θ_* to travel from the initial position to the target position in as short a distance as possible while avoiding collisions with one another.

The robots’ states at this moment were only determined by their states and decisions (actions) in the last moment. The set of states and actions were expressed as follows:

(5) $$Sd={\left\{{\left(s_{i}^{mT},a_{i}^{mT}\right)}_{m=0:k_{i}^{g}}\right\}}_{i=1,2,\ldots ,N}$$

where *Sd* represents the sequential decisions consisting of states and actions. Therefore, we could treat the above problem as a partially observable sequential decision-making problem, which can be formulated as a partially observable Markov decision process (POMDP) solved with reinforcement learning [4].

## 4. Algorithm Framework and Training

As mentioned above, we considered the problem as a POMDP and solved it by designing a reinforcement learning framework. A POMDP is formally defined by a seven-tuple (*S*, *A*, *Pr*, *R*, *O*, *Z*, *γ*), where *S* is the state space, *A* is the action space, *Pr* is the state transition function, *R* is the reward function, *γ* ∈ [0,1] is the discount factor, *O* is a finite set of observations (*o* ∈ *O*), and *Z* is an observation function (*o*~*Z*(*s*)). The state space *sokT* and the action space *akT* were described in Section 3. Next, we introduce the reward function in the reinforcement learning framework, network architecture, and training procedure.

### 4.1. Reward Design

The design of rewards is a key part of the reinforcement learning framework. In this section, we designed rewards for the optimization problems mentioned above. The reward function was designed as follows:

(6) $$r_{i}^{kT}={{(}^{g}r)}_{i}^{kT}+{{(}^{c}r)}_{i}^{kT}$$

where *r_i_^kT^* represents the reward of the *i*-th robot at time *kT*. (*^g^r*)*_i_^kT^* rewarded the robot to reduce the number of direction changes and to move toward the goal, and (*^c^r*)*_i_^kT^* rewarded the robot to prevent collisions.

When the robot reached the goal, (*^g^r*)*_i_^kT^* = *r_arrival_*. In order to reduce the number of direction changes, the value of (*^g^r*)*_i_^kT^* was designed as shown in Table 2, where *m_x_^kT^* or *m_y_^kT^* indicate, respectively, whether the robot was closer or farther from the target on the *x* or *y* axis at time (*k* − 1) *T* to *kT*. *w*_1_ and *w*_2_ (*w*_2_ > *w*_1_) were weighting factors.

(7) $$m_{x}^{kT}=\left\|p_{ix}^{(k-1)T}-g_{ix}^{}\right\|-\left\|p_{ix}^{kT}-g_{ix}^{}\right\|$$

(8) $$m_{y}^{kT}=\left\|p_{iy}^{(k-1)T}-g_{iy}^{}\right\|-\left\|p_{iy}^{kT}-g_{iy}^{}\right\|$$

The calculation of (*^c^r*)*_i_^kT^* was as follows:

(9) $$dis_{ij}^{kT}=\left\|p_{ix}^{kT}-p_{jx}^{kT}\right\|+\left\|p_{iy}^{kT}-p_{jy}^{kT}\right\|$$

(10) $${{(}^{c}r)}_{i}^{kT}=\{\begin{array}{c} \begin{array}{cc} r_{collision} & \begin{array}{cc} if & dis_{ij}^{kT}<D \end{array} \end{array}\\ \begin{array}{cc} 0 & \begin{array}{cc} & otherwise \end{array} \end{array} \end{array}$$

where *dis_ij_^kT^* represents the Manhattan distance between the *i*-th robot and the *j*-th robot at time *kT*. During the training of this work, we set *r_arrival_* as 1 and *r_collision_* as −1.

### 4.2. Network Architecture and Training Procedure

**Figure 3:**
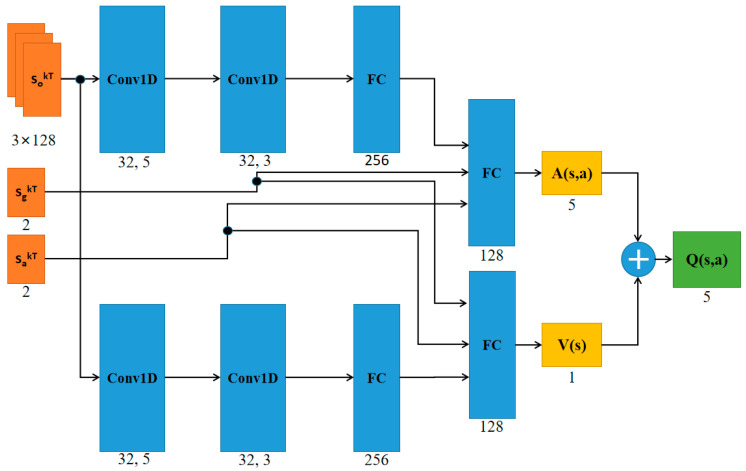
The structure diagram of the neural network. *s_o_^kT^*, *s_g_^kT^*, and *s_a_^kT^* were used as the input of the network structure, and the output of the network structure was the *Q* value of five actions. ReLU [32] was used as the non-linear activation function. Conv1D, convolutional neural network; FC, fully connected neural network.

The neural network mapped the input data *s* and the output *Q* (*s*, *a*) values (Figure 3). After the robot obtained the value of *Q* (*s*, *a*) in state *s*, it executed the action *a* with the highest *Q* (*s*, *a*) value.

**Figure 4:**
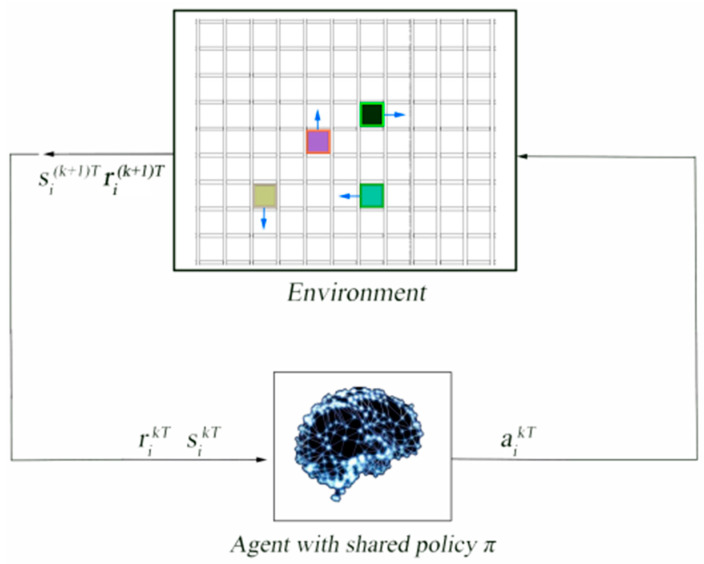
Overview of the process of data collection. At time *kT*, the *i*-th robot performed the action *a_i_^kT^* generated according to the policy *π* in state *s_i_^kT^*.

Figure 4 shows how each robot exploited the same policy to generate actions and interacted with the environment to get rewards and the next state. The obtained sets (*s_i_*^(*k*+1)*T*^, *a_i_^kT^*, *r_i_^kT^*, and *s_i_^kT^*) were stored in a fixed-size memory. Sampling by the parallel execution strategy was adopted in the training process. This method dramatically reduced the time of sample collection.

The training procedure, outlined in Algorithm 1, had two major steps: collecting data in parallel and updating policy. Double Deep Q-Network (DDQN) [33] was used in this process. The network was trained by back-propagation to minimize a quadratic regression error Re (Equation (12)) with the sample random batch of sets from the memory in every episode. Adaptive moment estimation (Adam) [34] was used as the optimization method of the network model training in this work.

(11) $$y_{k}=r^{kT}+\gamma Q(s^{{}_{(k+1)T}},\underset{a^{(k+1)T}}{\arg\max}Q(s^{{}_{(k+1)T}},a^{(k+1)T};\theta );\theta_{-})$$

(12) Re=(yk−Q(skT,akT;θ))2

where *θ_-_* represents the second set of weights.

**Algorithm 1:** DQN with Multiple Robots in Grid Map 1**Initialize replay memory *D* to capacity *Ca*;** 2**Initialize action-value function *Q* with random weights *θ*;** 3**Initialize target action-value function *Q* with weights *θ_* = *θ*;** 4**For** episode = 1, 2,... **do** 5  // Collect data in parallel 6  **For** robot *i* = 1, 2,..., *N*
**do** 7   Run *T_i_* timesteps 8   With probability ε select a random action *a_t_* 9   otherwise select *a_t_* = argmax*_a_ Q*(*ϕ*(*s_t_*), *a*; *θ*), where *t*∈[0, *T_i_*]10   Execute action *a_t_* emulator and observe reward *r_t_* and *s_t+1_*11   Preprocess *ϕ_t+1_* = *ϕ*(*s_t+1_*)12   Store transition (*ϕ_t_*, *a_t_*, *r_t_*, *ϕ_t+1_*) in *D*13   break, if $\sum_{i=1}^{N}T_{i}>T_{\max}$14  **End For**15  //*Updata policy*16  **if**
*the state in step j+1 is terminnated*
**then**17   *y_j_* = *r_i_*18  **else**19   *y_j_* = *r_i_* + *γmax_a’_*
*Q*(*ϕ_j+1_, a’*; *θ*_)20  **end**21  Perform a gradient descent step on (*y_j_*−*Q*(*ϕ_j_*, *a_j_*; *θ*))^2^ with respect to the network parameters *θ*22  Every *C* steps reset *Q* = *Q*23**End For**

## 5. Experiment

In this section, we describe the training scenario designed in this work and demonstrate that the learned policy had good performance in the grid map workspace.

### 5.1. Training Scenarios

**Figure 5:**
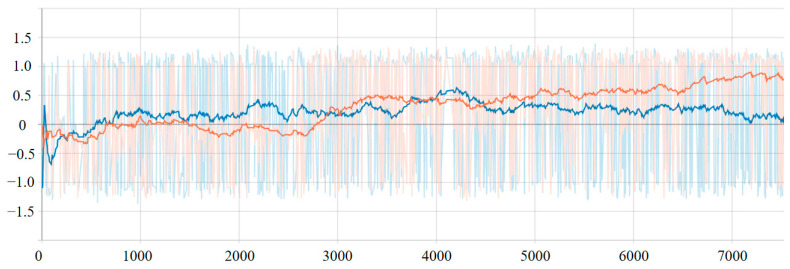
Reward curves during the training process; the orange curve is the training curve of Policy_1, and the blue curve is the training curve of Policy_2.

In this work, we used PyTorch to implement our algorithm. We designed training scenarios for four and eight robots in the grid map workspace based on the Stage (http://rtv.github.io/Stage/) mobile robot simulator. During the training process, the initial position and the goal were set within 16 × 16 of the domain size (without limiting the robots’ range of motion). In the training scene, each robot randomly generated initial and target positions. The algorithm proposed in this article was trained on a computer equipped with an E5-1620 CPU and an Nvidia GTX1060 GPU. The size of the mini-batches was set to 1024. The size of the fixed-size memory *D* was 2048 in this experiment. Step *C* in Algorithm 1 was 20. The algorithm used an ε-greedy policy, where ε decayed linearly from 0.5 to 0.3 in the first 3000 training episodes, and remained 0.3 thereafter. Using our proposed framework, Policy_1 was obtained by training the algorithm in the scenario of four robots, and Policy_2 was obtained by training the algorithm in the scenario of eight robots. Policy_1 and Policy_2 were trained on approximately 500,000 samples and 1,000,000 samples, respectively. Figure 5 shows the reward curves during the training process.

### 5.2. Simulation Results

To evaluate the policy learned by our proposed deep reinforcement learning framework, we designed random experimental scenarios.

In this scenario, the number of robots was gradually increased from two to nine. In the case of each quantity, the experiment was designed to complete 500 cases randomly generated with uniform distribution (the initial positions and goals of each robot were set within 16 × 16 of the domain size). When the robots were guided by policy (Policy_1 or Policy_2), traveling from the initial position to the goal position within a certain time and avoiding collisions with others while traveling, the case was completed successfully. Policy_1 and Policy_2 were tested by using 312,925 and 269,440 samples, respectively. Each case contained a different number of samples. Table 3 shows the success rate of the case and the number of samples used in the test.

When the robots were guided by Policy_1 or Policy_2 to complete the task and the number of robots in the scene was not more than three or five, respectively, there were no collisions during the experiment. When the number of robots in the scene was three or more, Policy_2 could guide robots to avoid collisions better than Policy_1. Therefore, it was concluded that the policy trained with more robots in the training scenario can better guide the robots to avoid collisions because deep reinforcement learning relies on interaction with the environment during the training process.

Since all of the states existing in the environment could not be encountered in the training process, the success rate slowly decreased as the number of robots in the scene increased. However, even if there were nine robots in the scenario, they were guided by Policy_1, which was learned from the scenario of four robots, to complete the task with a high success rate. Moreover, the hybrid control architecture proposed by [35] can be used to make up for this shortcoming, which found the emergent scenario based on the measurements of the Lidar sensor and designed a safe policy for this scenario. Combining the safety policy and the learned policy can achieve collision-free travel between robots.

### 5.3. Compared with Centralized Methods

The centralized methods, using a planning algorithm, produced a path that allowed a robot to avoid collisions with others. Although these methods had good performance, they relied heavily on the communication between the central server and the robots, and can be computationally prohibitive for large multi-robot systems.

**Figure 6:**
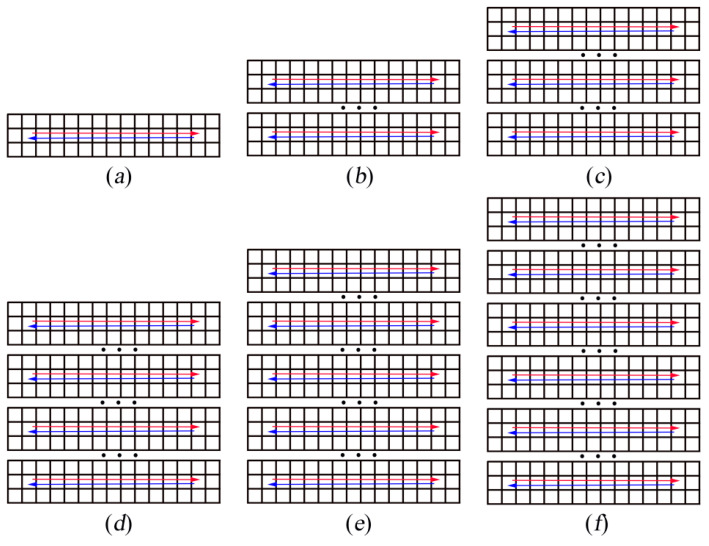
(**a**–**f**) Schematic diagrams of the start and end positions of the six cases. The position pointed to by the arrow is the end position of each robot. Three dots indicate that seven rows of grids were omitted.

**Figure 7:**
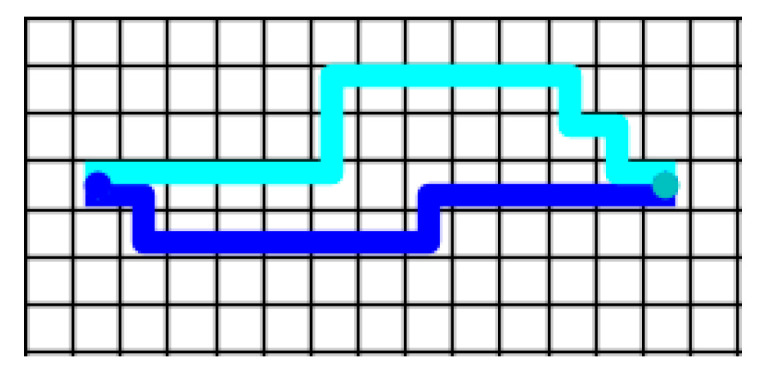
Robot trajectories using Policy_2 to guide the robot to complete the task in Figure 6a.

First, six cases were designed (Figure 6). Three centralized methods—Enhanced Partial Expansion A* (EPEA*) [25], Increasing Cost Tree Search (ICTS) [26], and Conflict-Based Search (CBS) [27]—and our policy were used to calculate the time required to complete the task path. The equipment used in this experiment was a computer with CPU E5-1620. For our policy, it took 9.5 ms for each robot to perform every step. Figure 7 shows the trajectory diagram of using Policy_2 to guide the robot to complete the task in Figure 6a. The two robots performed a total of 30 steps to complete the task, and it took 285 ms to calculate the trajectory using Policy_2. The experimental results are shown in Table 4.

**Figure 8:**
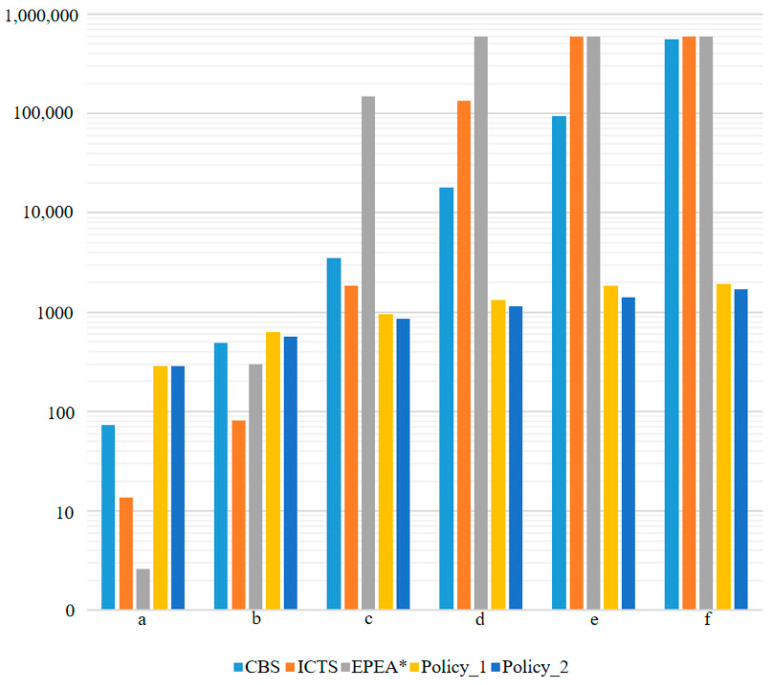
The time-consuming histogram use different methods to calculate the trajectory of the completed task shown in Figure 6.

Table 4 and Figure 8 show that in cases (c)–(f), the time consumed by Policy_1 and Policy_2 to calculate the trajectory was much lower than that of the centralized methods. As mentioned above, it was difficult for the centralized methods to calculate the trajectory of a large-scale multi-robot system. In addition, in cases (a) and (b), the time consumed was lower than our policy, but the centralized methods relied heavily on the communication between the central server and the robot. If the central server or communication system crashed, it would have caused the entire multi-robot system to crash. The policy we proposed does not require a central server and does not rely on communication, which greatly improves the robustness of the multi-robot system.

**Figure 9:**
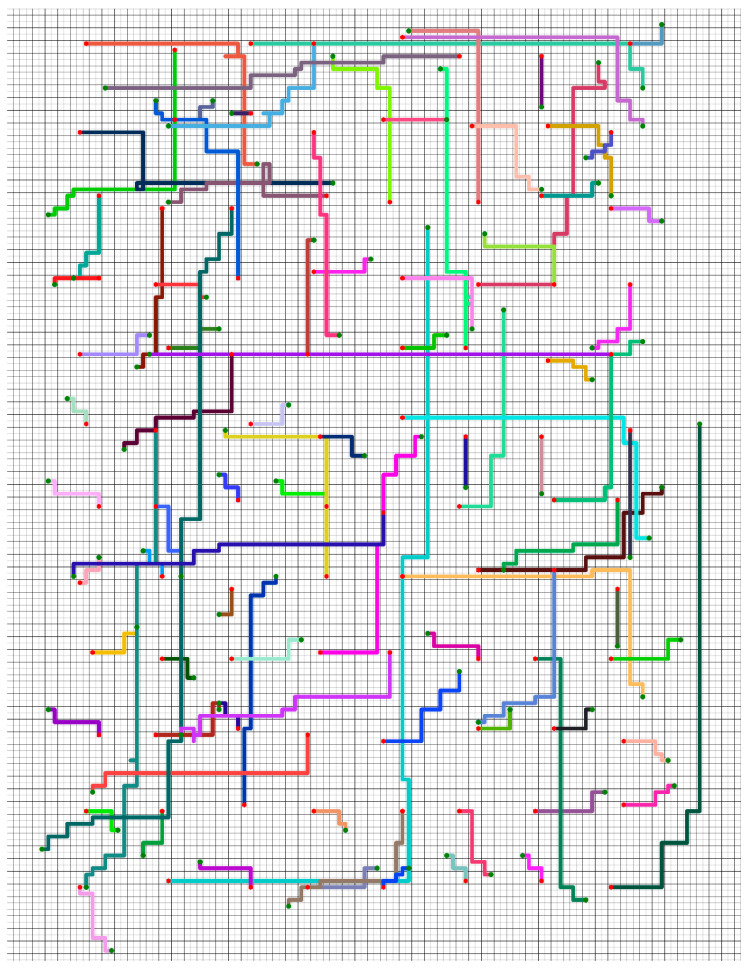
The trajectories of using Policy_2 to guide 96 robots to complete tasks in the large-scale grid map workspace. Different colors indicate the trajectories of different robots. The red hexagon is the initial position, and the green dot is the target position.

The policy we proposed can also guide robots to complete tasks in a large-scale grid map workspace. Figure 9 shows the trajectories of using Policy_2 to guide 96 robots to complete tasks in the large-scale grid map workspace (the initial and target positions of each robot were randomly generated).

We used *r_et_*, which represents the ratio of actual time to ideal time, as the evaluation algorithm performance metric, calculated as follows:

(13) $${\bar{r}}_{et}=\frac{1}{N}\sum_{i=1}^{N}\frac{k_{i}^{g}}{(\left\|p_{ix}^{0\cdot T}-g_{ix}\right\|+\left\|p_{iy}^{0\cdot T}-g_{iy}\right\|)/D}$$

where (*p_ix_*^0 *T*^, *p_iy_*^0 *T*^) represents the initial position of the *i*-th robot, and (*g_i_^x^*, *g_i_^y^*) represents the target position of the *i*-th robot.

**Figure 10:**
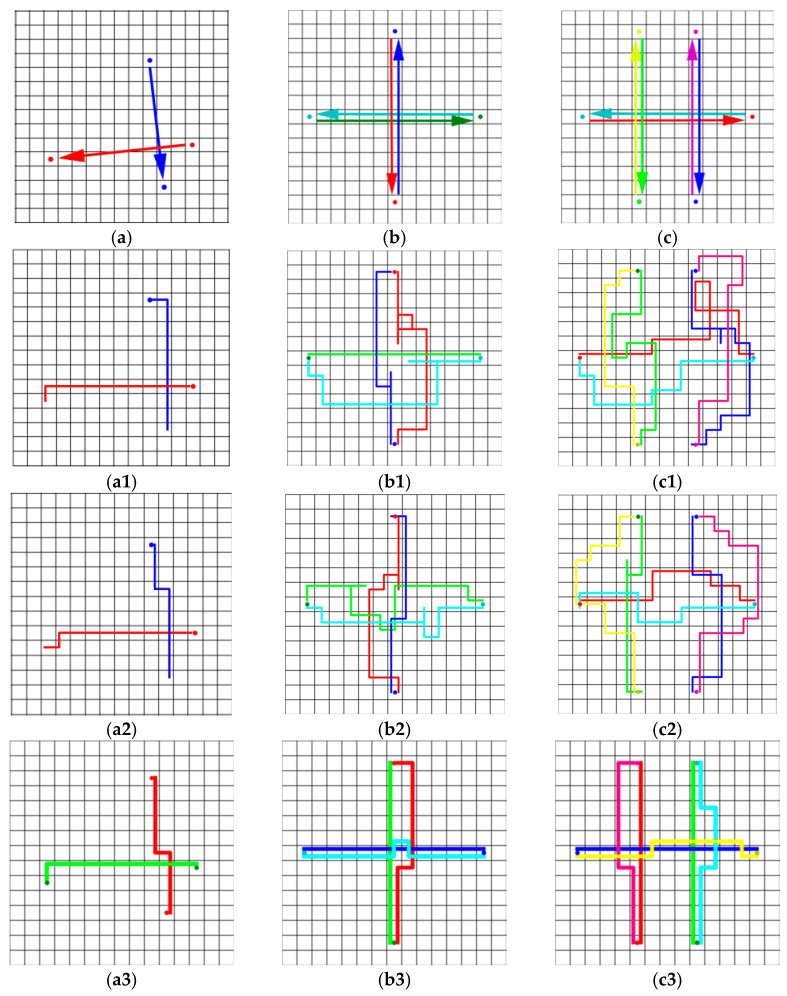
(**a**–**c**) Schematic diagrams of the start and end positions of the three cases. The position pointed to by the arrow is the end position of each robot. In cases (**b**,**c**), the robots gathered in the center. (**a1**–**c1**), (**a2**–**c2**), and (**a3**–**c3**) are the robot trajectories, which used different methods to guide the robot to complete the case, where hexagons mark the starts. The policies used in (**a1**–**c1**), (**a2**–**c2**), and (**a3**–**c3**) are Policy_1, Policy_2, and CBS respectively.

Three cases were designed to compare our strategy with CBS. Figure 10 shows the resulting trajectories, where *a*1, *a*2, and *a*3 in our policy had the same performance as the centralized methods in case *a*.

These cases were designed using *r_et_* to evaluate the performance of different methods (Table 5). The performance of CBS was better than our policy, especially in robot-intensive scenarios, because the learned policy focusing on local collision avoidance could not replace a global path planner. However, our policy did not rely on the communication between the robot and the central server, and was computationally feasible for large-scale multi-robot systems. In the case of dense robots, Policy_2 could guide robots to complete tasks better than Policy_1, such as in cases (b) and (c). As mentioned in the previous section, the policy trained with more robots in the training scenario could better guide the robots to avoid collisions.

## 6. Conclusions

We developed a novel decentralized multi-robot collision avoidance method with deep reinforcement learning, suitable for the large-scale grid map workspace multi-robot system that directly processes Lidar signals instead of communicating between the robots. The learned policy guided robots to complete tasks with a high success rate. Robots could be guided by the learned policy to complete tasks in a large-scale grid map workspace. Although the performance was lower than when using the centralized methods, the learned policy did not rely on communication with the central server. The method we proposed overcame the limitations of the distributed method (which cannot be used in the grid map workspace) and of centralized methods (which depends on communication and cannot be applied to the limitation of the large-scale grid map workspace). Future work will consider how to improve the performance of the method.

## Figures and Tables

**Table 1:** The correspondence between the last action and *s_a_^kT.^*

Last Action	Forward	Backward	Left	Right	Stop
***s_a_^kT^***	(0,1)	(0,−1)	(−1,0)	(1,0)	(0,0)

**Table 2:** The first part of the reward calculation.

*a^kT^* (*^g^r*)*_i_^kT^* *s_a_^kT^*	(0,1)	(0,−1)	(1,0)	(−1,0)
(0,v)	*w*_2_ × *m_y_^kT^*	*w*_2_ × *m_y_^kT^*	*w*_1_ × *m_y_^kT^*	*w*_1_ × *m_y_^kT^*
(0,−v)	*w*_2_ × *m_y_^kT^*	*w*_2_ × *m_y_^kT^*	*w*_1_ × *m_y_^kT^*	*w*_1_ × *m_y_^kT^*
(v,0)	*w*_1_ × *m_x_^kT^*	*w*_1_ × *m_x_^kT^*	*w*_2_ × *m_x_^kT^*	*w*_2_ × *m_x_^kT^*
(−v,0)	*w*_1_ × *m_x_^kT^*	*w*_1_ × *m_x_^kT^*	*w*_2_ × *m_x_^kT^*	*w*_2_ × *m_x_^kT^*

**Table 3:** Success rate of the cases with different numbers of robots.

Num	Policy_1		Policy_2	
2	500/500 (9910)	100%	500/500 (9385)	100%
3	500/500 (16,605)	100%	500/500 (15,460)	100%
4	493/500 (22,505)	98.60%	500/500 (21,895)	100%
5	487/500 (29,420)	97.40%	500/500 (27,250)	100%
6	486/500 (37,845)	97.20%	495/500 (36,580)	99.00%
7	483/500 (48,985)	96.60%	493/500 (42,145)	98.60%
8	483/500 (65,555)	96.60%	492/500 (52,000)	98.40%
9	479/500 (82,100)	95.80%	490/500 (64,725)	98.00%

The success rate of the different policies (Policy_1 and Policy_2) for completing cases in random scenarios. Among them, in the form of 493/500 (22,505), denominator represents the number of successfully completed cases, molecular represents the total number of tested cases, and the number in brackets represents the total number of tested samples.

**Table 4:** Time consumption, in milliseconds (ms). CBS, Conflict-Based Search; ICTS, Increasing Cost Tree Search; EPEA*, Enhanced Partial Expansion A*.

Policy	a	b	c	d	e	f
CBS	73.2	493.1	3545.8	18,168.3	94,618.5	564,439.2
ICTS	13.58	81.75	1860	135,365	>600,000	>600,000
EPEA*	2.61	299.3	148,224.7	>600,000	>600,000	>600,000
Policy_1	285	627	959.5	1339.5	1862	1947.5
Policy_2	285	570	855	1140	1425	1710

**Table 5:** The performance metric in the different cases.

Cases	CBS	Policy_1	Policy_2
a	1.0	1.000	1.000
b	1.104	1.917	1.479
c	1.084	1.986	1.514

## Data Availability

The data presented in this study are available on request from the corresponding author.

## Abbreviations

The following abbreviations are used in this manuscript:

DDQN	Double Deep Q-Network
Adam	Adaptive moment estimation
Conv1D	convolutional neural network
FC	fully connected neural network
EPEA*	Enhanced Partial Expansion A*
ICTS	Increasing Cost Tree Search
CBS	Conflict-Based Search
CADRL	collision avoidance with deep reinforcement learning
RVO	reciprocal velocity obstacle
ORCA	optimal reciprocal collision avoidance
GA3C-CADRL	GPU/CPU Asynchronous Advantage Actor-Critic for Collision Avoidance with Deep reinforcement learning
SA-CADRL	socially aware CADRL
